# Prevalence of preconception TORCH infections and its influential factors: evidence from over 2 million women with fertility desire in southern China

**DOI:** 10.1186/s12905-023-02560-4

**Published:** 2023-08-10

**Authors:** Lu Han, Rui Li, Wenxue Xiong, Yang Hu, Jiabao Wu, Xiaohua Liu, Hua Nie, Weibing Qin, Li Ling, Mingzhen Li

**Affiliations:** 1 NHC Key Laboratory of Male Reproduction and Genetics, Guangdong Provincial Reproductive Science Institute (Guangdong Provincial Fertility Hospital), 510600 Guangzhou, China; 2 Faculty of Medical Statistics, School of Public Health, Sun Yat-sen University, 510080 Guangzhou, Guangdong China; 3 Clinical research design division, Clinical research center, Sun Yat-sen Memorial Hospital, Sun Yat-sen University, Guangzhou, Guangdong China

**Keywords:** Toxoplasma gondii, Cytomegalovirus, Rubella virus, Seroprevalence, Influential factor

## Abstract

**Background:**

TORCH (Toxoplasma gondii [TOX], Cytomegalovirus [CMV], Rubella virus [RV], and Herpes simplex virus [HSV]) represents pathogens known to traverse the maternal-fetal barrier and cause severe neonatal anomalies. We aimed to assess the prevalence of preconception TOX, CMV, and RV infections among women with fertility desire in southern China, and identify related risk factors.

**Methods:**

Data were obtained from a population-based cross-sectional study conducted as part of the National Free Preconception Health Examination Project. Women planning to conceive within the next 6 months in Guangdong Province were enrolled between 2014 and 2019. Information on sociodemographic, gynecological, and obstetric characteristics was collected. Sera were analyzed for TOX IgG, CMV IgG, and RV IgG antibodies using an enzyme-linked immunosorbent assay. Descriptive, univariate, and multivariate logistic regression analyses were performed to assess the association between TORCH infections and related factors.

**Results:**

Among 2,409,137 participants, the prevalence of IgG antibodies for TOX, CMV, and RV was 3.20% (95% *CI*: 3.18–3.22%), 77.67% (95% *CI*: 77.62–77.71%) and 76.03% (95% *CI*: 75.98–76.07%), respectively. Of all participants, 141,047 women (5.85%, 95% *CI*:5.83–5.88%) reported a history of immunization for RV. Women living in the Pearl River Delta, a more developed region, have significantly lower vaccination rates than those living in other regions. The seropositivity of TOX IgG was highest among women aged 35 years and above, with primary or lower education levels, and rural registration. Factors such as being older, having a higher educational level, and being of other ethnicities were associated with a higher prevalence of naturally acquired CMV and RV infections. Women living in the Pearl River Delta showed a higher risk of TOX, CMV, and RV infections, with a*OR*s of 2.21, 4.45, and 1.76, respectively. A history of pregnancy, gynecological diseases, and sexually transmitted infections were potentially associated with TORCH infections, but this association varied across pathogens.

**Conclusion:**

The findings of this study update the baseline of preconception TORCH infections among women with fertility desire in southern China, helping to estimate the risk of congenital infection and guide the development and implementation of effective prevention measures for preconception TORCH infections.

**Supplementary Information:**

The online version contains supplementary material available at 10.1186/s12905-023-02560-4.

## Background

TORCH represents a series of pathogens known to traverse the maternal-fetal barrier and cause adverse pregnancy outcomes and congenital infection in the fetus, conventionally including Toxoplasma gondii (TOX), Cytomegalovirus (CMV), Rubella virus (RV), and Herpes simplex virus (HSV) [1, 2]. Globally, 2–3% of congenital anomalies are attributable to TORCH infections during pregnancy [3]. The annual burden of disability-adjusted life years (DALYs) related to congenital toxoplasmosis is estimated to be 1.20 million DALYs [4]. Approximately 1 million cases of congenital rubella syndrome (CRS) are estimated to occur every year [5], and the case fatality ratio for CRS ranges from 5 to 34%, contributing to approximately 5,000–34,000 annual deaths [6, 7]. Studies have demonstrated that acquiring TORCH infections during the first trimester of gestation is associated with the highest risk of severe neonatal complications [7, 8], necessitating early screening before the critical period of fetal organogenesis [9, 10]. Preconception screening for reproductive-aged women, especially those with a desire for fertility, helps to offer prompt diagnosis and timely treatment decisions for infected women and to carry out prevention measures to reduce primary TORCH infections during pregnancy.

The prevalence of TORCH infections among reproductive-aged women varies dramatically worldwide, with TOX infections ranging from approximately 10% in Switzerland and the United States to over 60% in Iran and Indonesia [11], and CMV infections ranging from less than 50% in Ireland, France, and the United Kingdom to over 90% in Turkey and Korea [12, 13]. In China, one of the most populous countries, the estimated national seroprevalences of TOX, CMV, and RV IgG antibodies in 2010 and 2012 were 2.3% [14], 38.6% [15], and 58.4% [16], respectively. However, the burden of TORCH among women of reproductive age may be underestimated because of the asymptomatic nature of TORCH infections and insufficient screening programs. Prior studies in China have mainly focused on rural residents [17], but the increasing concentration and mobility of the population in urban areas process might facilitate the TORCH transmission among urban residents [18, 19]. Seroepidemiological data on TORCH infections in both rural and urban areas are urgently needed to estimate the infection risk more extensively and precisely. In addition, understanding the potential risk factors for acquiring TORCH infections is important for improving primary prevention strategies. Emerging evidence suggests that the prevalence of TORCH infections may be associated with demographic and clinical characteristics, such as age, ethnicity, educational level, and history of pregnancy [20–22], but the significance and directionality of these associations are not consistent [23]. Thus, the factors that influence TORCH infections need to be clarified.

The National Free Preconception Health Examination Project (NFPHEP) conducted in Guangdong Province has provided free preconception screening for TOX, CMV, and RV infections in women desiring fertility in both urban and rural areas. This offered us the opportunity to comprehensively understand the epidemiological profile of TORCH infections in a province with the largest population in southern China (115 million in 2019, including 22.4 million married women) [24] and unevenly distributed medical resources. Therefore, using more than 2.4 million samples derived from the NFPHEP in Guangdong from 2014 to 2019, we determined the prevalence of TOX, CMV, and RV infection among women with fertility desire and explored its influential factors combined with information on sociodemographic, gynecological, and obstetric characteristics. The findings of this study could update the baseline estimates of these infections and guide the development and implementation of effective prevention measures, particularly among vulnerable groups.

## Materials and methods

### Study design and participants

The NFPHEP is a nationwide population-based cross-sectional survey conducted since 2010 by the Chinese National Health and Family Planning Commission and the Finance Ministry in mainland China. This project provided free preconception care services, including health examinations, risk evaluations, and medical consultations, for married couples planning to conceive within six months. The government of Guangdong province actively responded and expanded the target population to include both rural and urban married couples with the goal of reducing the potential risk of birth defects and improving maternal health. More details about the design, organization, implementation, and quality control of this project have been described previously [25, 26]. Before enrolment, all the participants provided written informed consent. This study was approved by the Institutional Review Board of the Chinese Association of Maternal and Child Health Studies (IRB-201,001).

In this study, we extracted Guangdong data from the NFPHEP database regarding the physical examination and individual basic information of 2,626,851 women of reproductive age (21–49 years) from 2014 to 2019. Participants with incomplete information regarding the serostatus of TOX, CMV, and RV IgG antibodies and duplicate records were excluded from the analysis. Finally, data from 2,409,137 women were used for statistical analyses. A flowchart of the study population selection is shown in **Supplementary Fig. 1**.

### Outcome and variables

Venous blood (5 mL) was collected from each participant and immediately sent to qualified local laboratories, where samples were stored at -30 °C before being tested. Specific IgG antibodies against TOX, CMV, and RV were tested in local laboratories affiliated with medical institutions under qualified quality control mechanisms with available enzyme-linked immunosorbent assay (ELISA) kits according to the manufacturer’s instructions. The reagent kits were approved by the China Food and Drug Administration and selected by local laboratories based on their preference. Infection status was defined based on the results of testing for TOX IgG, CMV IgG, and RV IgG antibodies. Women showing IgG antibody positivity were considered to have latent infections, and those showing IgG antibody negativity were considered susceptible to the pathogen. Participants with suspicious test results and requiring further diagnostic examination were considered negative for IgG antibodies.

**Fig. 1:**
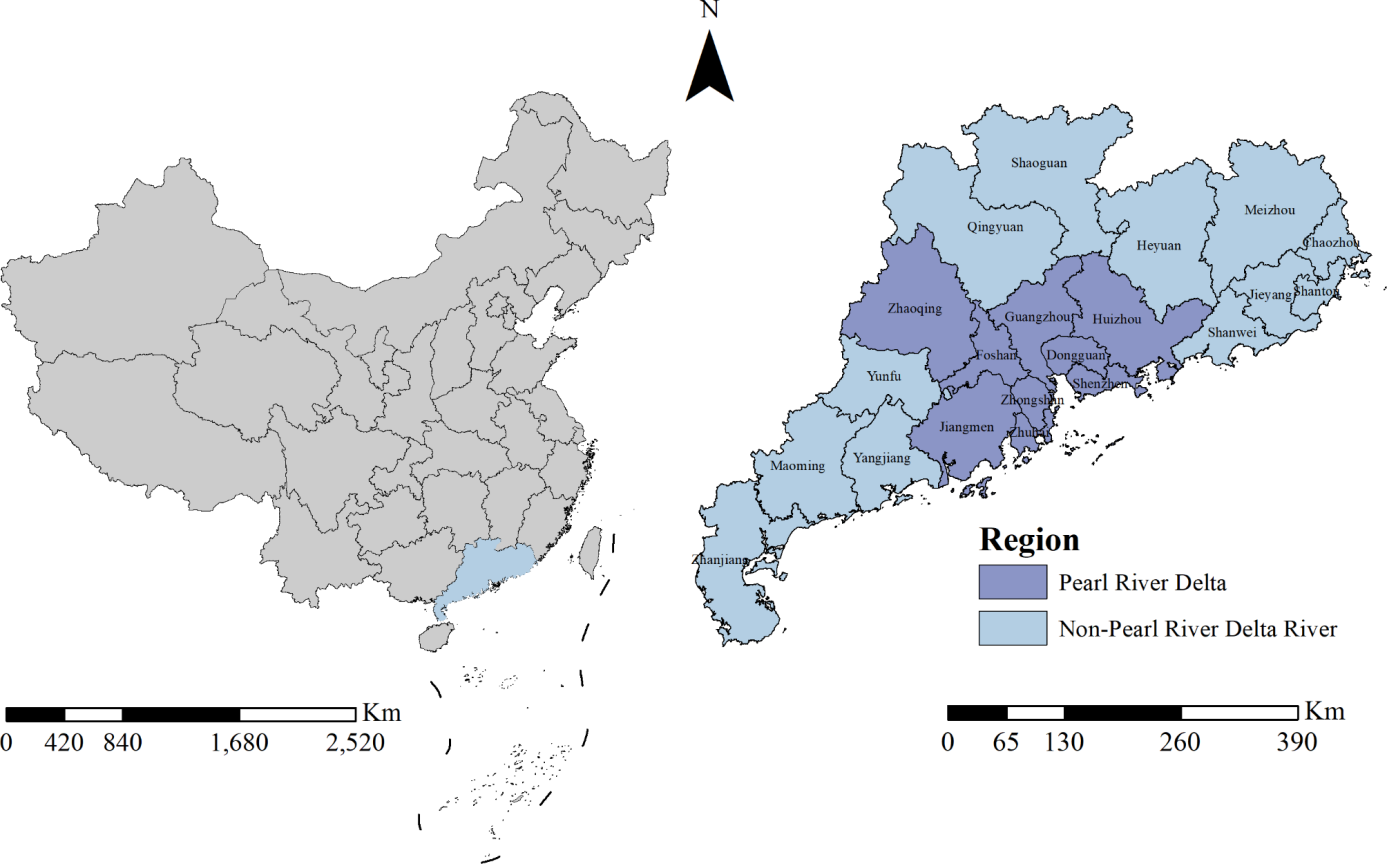
Location of Guangdong province in China and the economic geographical division of Guangdong

Information on sociodemographic characteristics (age, education level, occupation, ethnicity, and household registration), history of pregnancy, gynecological diseases, and sexually transmitted infections (STIs) was collected by a locally trained health worker using a standardized questionnaire. At the same time, the history of RV vaccination was recorded based on the participant’s answer to a “yes or no” question, namely “Have you ever been vaccinated against RV?”. The ages of the women who participated in this study were grouped: 21–24, 25–29, 30–34, 35–39, 40–44, and 45–49 years old. Educational levels were divided into primary school or below, junior high school, senior high school, and college or higher. Occupations were classified as farmers, workers, or other. Ethnicity was grouped into Han and other ethnic groups. The household registration of each participant was classified as rural or urban. In addition, the economy in Guangdong province developed rapidly but unevenly, and the Pearl River Delta region contributed almost 80% of the GDP of the entire province. Thus, the socioeconomic status (SES) of the study participants was measured by residential addresses and divided into living in the Pearl River Delta region (Guangzhou, Foshan, Zhaoqing, Shenzhen, Dongguan, Huizhou, Zhuhai, Zhongshan, and Jiangmen) and non-Pearl River Delta regions (Heyuan, Meizhou, Qingyuan, Shaoguan, Yunfu, Shanwei, Shantou, Chaozhou, Jieyang, Zhanjiang, Yangjiang, and Maoming) (Fig. 1).

### Statistical analysis

These proportions were used to describe participants’ sociodemographic characteristics. The trends and spatial distribution of the prevalence of TOX, CMV, and RV infections from 2014 to 2019 are described. The prevalence of TOX, CMV, and RV IgG antibodies and their 95% confidence intervals (95% *CI*) were calculated for the entire study group and subpopulations with different individual characteristics. Crude odds ratios (c*OR*) and adjusted odds ratios (a*OR*) with 95% *CI* were calculated using univariate and multivariate logistic regression, respectively, to identify factors associated with the prevalence of TOX, CMV, and RV infections. Statistical significance was defined as a two-sided *P* value < 0.05. All analyses were performed using R version 4.0.3.

## Results

### Sociodemographic characteristics and prevalence of TOX, CMV, and CV IgG antibodies

**Fig. 2:**
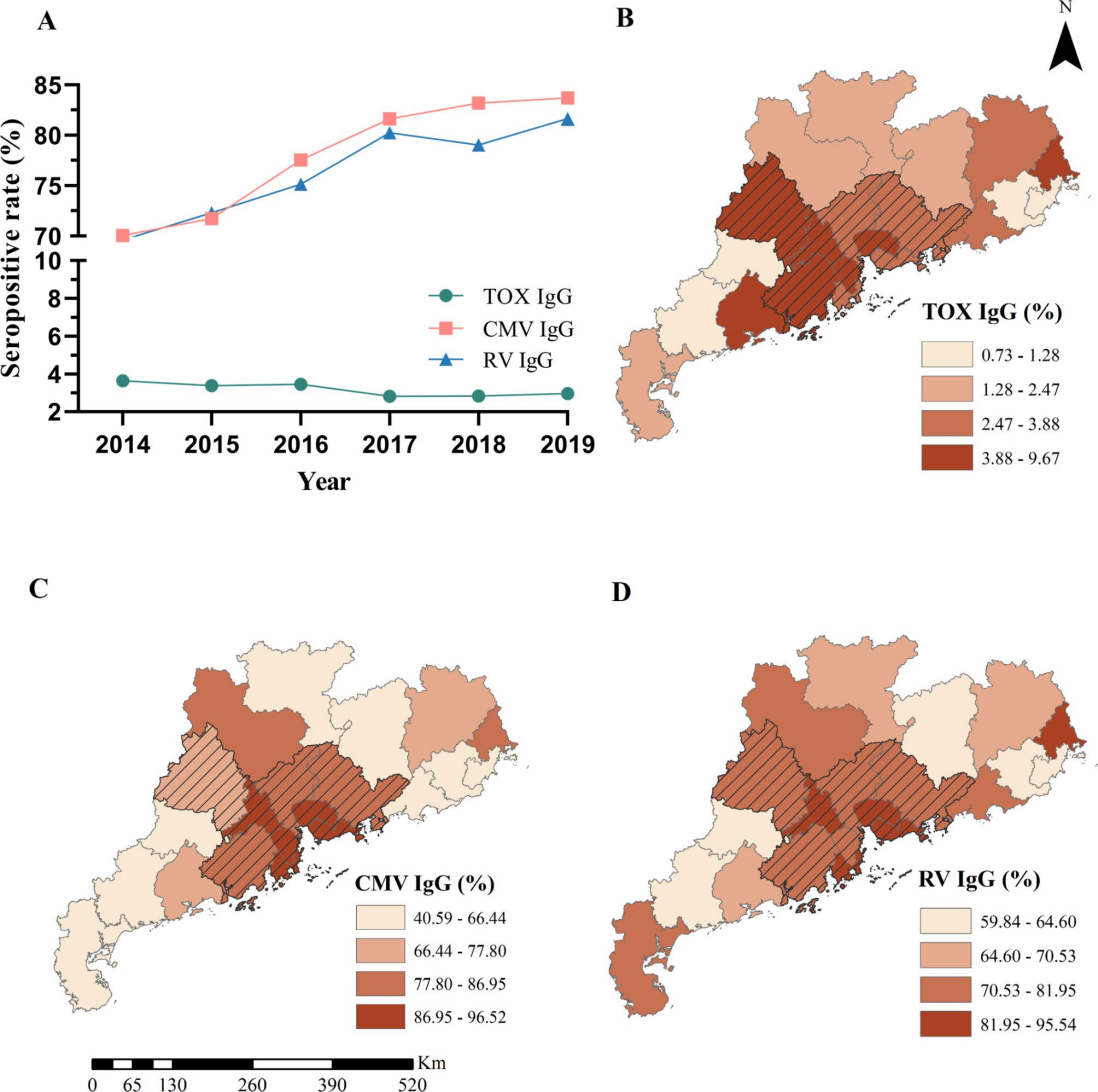
The prevalence trend and spatial distribution of TOX, CMV, and RV infections. **A**) The trend of seropositive rate of IgG antibodies for TOX, CMV, and RV among women with fertility desire in Guangdong from 2014 to 2019. **B**) The spatial distribution of TOX IgG antibodies. **C**) The spatial distribution of CMV IgG antibodies. **D**) The spatial distribution of RV IgG antibodies. The slash part represents the Pearl River Delta region. Abbreviation: TOX: Toxoplasma gondii; CMV: Cytomegalovirus; RV: Rubella virus; IgG: Immunoglobulin G

The detailed sociodemographic characteristics of the 2,409,137 women with fertility desires included in this study are shown in Table 1. The median age was 27 years (Interquartile Range: 24–30 years), the majority were of Han ethnicity (95.43%), and had a rural household registration (73.67%). A subgroup of 2,268,090 (94.2%) women who reported no history of RV vaccination was included in the analysis to investigate the association of individual characteristics with RV infection using natural methods. Their characteristics are shown in **Supplementary Table 1**. Of all participants in this study, the overall seropositivity of TOX, CMV, and RV IgG antibodies among women with fertility desire in Guangdong were 3.20% (95% *CI*:3.18–3.22%), 77.67% (95% *CI*:77.62–77.71%), and 76.03% (95% *CI*:75.98–76.07%), respectively. The annual seropositivity rates of CMV IgG and RV IgG antibodies increased slightly during the study period, whereas that of TOX IgG antibodies remained stable (Fig. 2A). The seropositivity of IgG antibodies varied dramatically across the cities (Fig. 2B-D). The high-prevalence cities were mainly concentrated in the Pearl River Delta region, which has a high population density and relatively high economic development level.

**Table 1:** Characteristics of 2,409,137 reproductive-aged women preparing for pregnancy, Guangdong, 2014–2019, *n* (%)

	2014–2019	2014	2015	2016	2017	2018	2019
All participants	2,409,137	427,336	385,081	490,260	438,856	384,900	282,704
Age groups, years							
21–24	626,377 (26.00)	144,003 (33.70)	123,545 (32.08)	108,214 (22.07)	104,146 (23.73)	87,125 (22.64)	59,344 (20.99)
25–29	1,092,742 (45.36)	200,722 (46.97)	190,473 (49.46)	191,078 (38.97)	192,079 (43.77)	181,196 (47.08)	137,194 (48.53)
30–34	430,953 (17.89)	60,621 (14.19)	51,898 (13.48)	97,531 (19.89)	84,679 (19.30)	76,148 (19.78)	60,076 (21.25)
35–39	179,508 (7.45)	17,430 (4.08)	14,472 (3.76)	64,078 (13.07)	38,331 (8.73)	26,944 (7.00)	18,253 (6.46)
40–44	63,203 (2.62)	4056 (0.95)	4011 (1.04)	25,036 (5.11)	14,834 (3.38)	9663 (2.51)	5603 (1.98)
45–49	16,354 (0.68)	504 (0.12)	682 (0.18)	4323 (0.88)	4787 (1.09)	3824 (0.99)	2234 (0.79)
**Education**							
Primary school or below	50,775 (2.11)	12,905 (3.02)	9101 (2.36)	9494 (1.94)	8292 (1.89)	6958 (1.81)	4025 (1.42)
Junior high school	678,031 (28.14)	160,153 (37.48)	124,436 (32.31)	132,237 (26.97)	117,319 (26.73)	84,238 (21.89)	59,648 (21.10)
Senior high school	572,328 (23.76)	104,005 (24.34)	90,632 (23.54)	105,414 (21.50)	106,495 (24.27)	95,656 (24.85)	70,126 (24.81)
College or higher	825,075 (34.25)	121,446 (28.42)	126,526 (32.86)	181,223 (36.96)	147,712 (33.66)	139,151 (36.15)	109,017 (38.56)
Not available	282,928 (11.74)	28,827 (6.75)	34,386 (8.93)	61,892 (12.62)	59,038 (13.45)	58,897 (15.30)	39,888 (14.11)
**Occupation**							
Farmers	499,342 (20.73)	126,492 (29.60)	91,583 (23.78)	97,638 (19.92)	80,077 (18.25)	63,373 (16.46)	40,179 (14.21)
Workers	542,027 (22.50)	98,298 (23.00)	86,772 (22.53)	102,736 (20.96)	105,922 (24.14)	82,397 (21.41)	65,902 (23.31)
Others	1,004,148 (41.68)	165,633 (38.76)	161,900 (42.04)	217,013 (44.26)	178,366 (40.64)	159,307 (41.39)	121,929 (43.13)
Not available	363,620 (15.09)	36,913 (8.64)	44,826 (11.64)	72,873 (14.86)	74,491 (16.97)	79,823 (20.74)	54,694 (19.35)
**Ethnicity**							
Han	2,299,010 (95.43)	415,619 (97.26)	370,855 (96.31)	467,548 (95.37)	417,507 (95.14)	361,536 (93.93)	265,945 (94.07)
Others	22,152 (0.92)	3973 (0.93)	3616 (0.94)	4059 (0.83)	3898 (0.89)	3698 (0.96)	2908 (1.03)
Not available	87,975 (3.65)	7744 (1.81)	10,610 (2.76)	18,653 (3.80)	17,451 (3.98)	19,666 (5.11)	13,851 (4.90)
**Household registration**							
Rural	1,774,759 (73.67)	332,681 (77.85)	295,517 (76.74)	339,222 (69.19)	317,466 (72.34)	279,323 (72.57)	210,550 (74.48)
Urban	632,327 (26.25)	94,652 (22.15)	89,490 (23.24)	149,835 (30.56)	120,619 (27.48)	105,577 (27.43)	72,154 (25.52)
Not available	2051 (0.09)	3 (0.00) ^a^	74 (0.02)	1203 (0.25)	771 (0.18)	0 (0.00)	0 (0.00)
**Region**							
Non-Pearl River Delta	1,195,970 (49.64)	224,321 (52.49)	196,824 (51.11)	234,980 (47.93)	224,072 (51.06)	184,267 (47.87)	131,506 (46.52)
Pearl River Delta	1,213,167 (50.36)	203,015 (47.51)	188,257 (48.89)	255,280 (52.07)	214,784 (48.94)	200,633 (52.13)	151,198 (53.48)

NFPHEP: National Free Preconception Health Examination Project

^a^ The proportion is less than 0.01 and has been rounded

### Associations with sociodemographic, gynecological, and obstetric characteristics

Table 2 summarizes the association between sociodemographic, gynecological, and obstetric characteristics and the prevalence of TOX IgG antibodies in both uni- and multivariate models. Compared with women aged 21–24 years, women aged 25–29 and 30–34 years were significantly less likely to be infected with TOX, with a*OR*s (95% *CI*) of 0.91 (0.89–0.93) and 0.97 (0.95-1.00), respectively. Women aged 35–39, 40–44, and 45–49 years were significantly more likely to be infected, with adjusted *OR*s (95% *CI*) as 1.19 (1.15–1.23), 1.39 (1.33–1.46), and 1.49 (1.38–1.62), respectively. When comparing the educational level of primary school or below, women with the educational level of junior high school, senior high school, and college or higher were less likely to be infected with TOX. Urban participants had a significantly lower risk of TOX infection than rural participants (a*OR* = 0.70, 95% *CI*:0.69–0.72). Women who were living in the Pearl River Delta region had a higher chance to be infected than those living the non-Pearl River Delta region (a*OR* = 2.21, 95% *CI*:2.17–2.25). Women with pregnancy experience had a greater risk of TOX infection compared with those without the experience (a*OR* = 1.12, 95% *CI*:1.10–1.14).

**Table 2:** Association between sociodemographic, gynecological, and obstetric characteristics and the prevalence of TOX IgG antibodies

Characteristics	Total	Positive	Seropositivity (95% *CI*)	c*OR* (95% *CI*)	a*OR* (95% *CI*)
**All participants**	2,409,137	77,112	3.20 (3.18–3.22)	-	-
**Age groups, years**					
21–24	626,377	19,005	3.03 (2.99–3.08)	1.00	1.00
25–29	1,092,742	31,853	2.91 (2.88–2.95)	0.96 (0.94–0.98)	0.91 (0.89–0.93)
30–34	430,953	14,771	3.43 (3.37–3.48)	1.13 (1.11–1.16)	0.97 (0.95-1.00)
35–39	179,508	7594	4.23 (4.14–4.32)	1.41 (1.37–1.45)	1.19 (1.15–1.23)
40–44	63,203	3022	4.78 (4.62–4.95)	1.60 (1.54–1.67)	1.39 (1.33–1.46)
45–49	16,354	867	5.30 (4.96–5.66)	1.79 (1.67–1.92)	1.49 (1.38–1.62)
**Education**					
Primary school or below	50,775	1610	3.17 (3.02–3.33)	1.00	1.00
Junior high school	678,031	20,469	3.02 (2.98–3.06)	0.95 (0.90-1.00)	0.90 (0.85–0.95)
Senior high school	572,328	20,377	3.56 (3.51–3.61)	1.13 (1.07–1.19)	0.96 (0.91–1.01)
College or higher	825,075	26,943	3.27 (3.23–3.30)	1.03 (0.98–1.08)	0.86 (0.82–0.91)
**Occupation**					
Farmers	499,342	14,730	2.95 (2.90-3.00)	1.00	1.00
Workers	542,027	18,735	3.46 (3.41–3.50)	1.18 (1.15–1.20)	1.01 (0.99–1.03)
Others	1,004,148	33,065	3.29 (3.26–3.33)	1.12 (1.10–1.14)	0.96 (0.94–0.98)
**Ethnicity**					
Han	2,299,010	73,680	3.20 (3.18–3.23)	1.00	1.00
Others	22,152	831	3.75 (3.50–4.01)	1.18 (1.10–1.26)	1.03 (0.96–1.11)
**Household registration**					
Rural	1,774,759	58,672	3.31 (3.28–3.33)	1.00	1.00
Urban	632,327	18,320	2.90 (2.86–2.94)	0.87 (0.86–0.89)	0.70 (0.69–0.72)
**Region**					
Non-Pearl River Delta	1,195,970	25,524	2.13 (2.11–2.16)	1.00	1.00
Pearl River Delta	1,213,167	51,588	4.25 (4.22–4.29)	2.04 (2.01–2.07)	2.21 (2.17–2.25)
**History of pregnancy**					
No	1,436,837	41,169	2.87 (2.84–2.89)	1.00	1.00
Yes	960,077	35,307	3.68 (3.64–3.71)	1.29 (1.28–1.31)	1.12 (1.10–1.14)
**History of gynecological diseases**					
No	2,312,194	72,792	3.15 (3.13–3.17)	1.00	1.00
Yes	79,839	3242	4.06 (3.92–4.20)	1.30 (1.26–1.35)	1.04 (1.00-1.08)
**History of STIs**					
No	2,405,509	76,958	3.20 (3.18–3.22)	1.00	1.00
Yes	3628	154	4.24 (3.61–4.95)	1.34 (1.14–1.58)	1.07 (0.90–1.28)

The prevalence of CMV IgG antibodies increased with age (Table 3). Women with an educational level of senior high school or higher had a higher risk of being infected than those with an educational level of primary school or lower. The prevalence of CMV IgG was significantly higher in ethnic minorities than in the Han population (85.69% vs. 77.56%, a*OR* = 1.44, 95% *CI*:1.38–1.50). Women who had urban household registration were at higher CMV infection risk than those with rural household registration (85.55% vs. 74.83%, a*OR* = 1.02, 95% *CI*:1.01–1.03). In addition, women with a history of pregnancy, gynecological diseases, or STIs had a significantly greater risk (a*OR*s [95% *CI*]:1.23 [1.22–1.24], 1.23 [1.20–1.26], 1.29 [1.13–1.47], respectively) than those who did not.

**Table 3:** Association between sociodemographic, gynecological, and obstetric characteristics and the prevalence of CMV IgG antibodies

Characteristics	Total	Positive	Seropositivity (95% *CI*)	c*OR* (95% CI)	a*OR* (95% CI)
**All participants**	2,409,137	1,871,093	77.67 (77.62–77.71)	-	-
**Age groups, years**					
21–24	626,377	441,856	70.54 (70.45–70.64)	1.00	1.00
25–29	1,092,742	846,819	77.49 (77.43–77.56)	1.44 (1.43–1.45)	1.06 (1.05–1.07)
30–34	430,953	359,299	83.37 (83.27–83.47)	2.09 (2.07–2.11)	1.15 (1.14–1.17)
35–39	179,508	154,398	86.01 (85.85–86.17)	2.57 (2.53–2.61)	1.23 (1.20–1.25)
40–44	63,203	54,473	86.19 (85.92–86.46)	2.61 (2.55–2.67)	1.26 (1.23–1.30)
45–49	16,354	14,248	87.12 (86.60-87.63)	2.83 (2.70–2.96)	1.27 (1.20–1.34)
**Education**					
Primary school or below	50,775	34,542	68.03 (67.62–68.44)	1.00	1.00
Junior high school	678,031	465,624	68.67 (68.58–68.76)	1.03 (1.01–1.05)	0.93 (0.91–0.95)
Senior high school	572,328	442,107	77.25 (77.15–77.34)	1.60 (1.56–1.63)	1.07 (1.04–1.09)
College or higher	825,075	710,581	86.12 (86.05–86.19)	2.92 (2.86–2.97)	1.25 (1.22–1.28)
**Occupation**					
Farmers	499,342	338,841	67.86 (67.75–67.96)	1.00	1.00
Workers	542,027	410,790	75.79 (75.69–75.89)	1.48 (1.47–1.50)	0.93 (0.92–0.93)
Others	1,004,148	838,595	83.51 (83.45–83.58)	2.40 (2.38–2.42)	1.12 (1.11–1.13)
**Ethnicity**					
Han	2,299,010	1,783,193	77.56 (77.52–77.61)	1.00	1.00
Others	22,152	18,982	85.69 (85.22–86.15)	1.73 (1.67–1.80)	1.44 (1.38–1.50)
**Household registration**					
Rural	1,774,759	1,328,129	74.83 (74.78–74.89)	1.00	1.00
Urban	632,327	540,977	85.55 (85.47–85.63)	1.99 (1.98–2.01)	1.02 (1.01–1.03)
**Region**					
Non-Pearl River Delta	1,195,970	780,571	65.27 (65.20-65.34)	1.00	1.00
Pearl River Delta	1,213,167	1,090,522	89.89 (89.84–89.94)	4.73 (4.70–4.77)	4.45 (4.41–4.49)
**History of pregnancy**					
No	1,436,837	1,073,980	74.75 (74.68–74.81)	1.00	1.00
Yes	960,077	787,765	82.05 (81.98–82.12)	1.54 (1.53–1.55)	1.23 (1.22–1.24)
**History of gynecological diseases**					
No	2,312,194	1,786,223	77.25 (77.20–77.30)	1.00	1.00
Yes	79,839	70,523	88.33 (88.11–88.55)	2.23 (2.18–2.28)	1.23 (1.20–1.26)
**History of STIs**					
No	2,405,509	1,867,858	77.65 (77.60–77.70)	1.00	1.00
Yes	3628	3235	89.17 (88.11–90.16)	2.37 (2.13–2.63)	1.29 (1.13–1.47)

CMV: Cytomegalovirus; STIs: Sexually transmitted infections; *CI*: Confidence interval; c*OR*: Crude odds ratio; a*OR*: Adjusted odds ratio

**Fig. 3:**
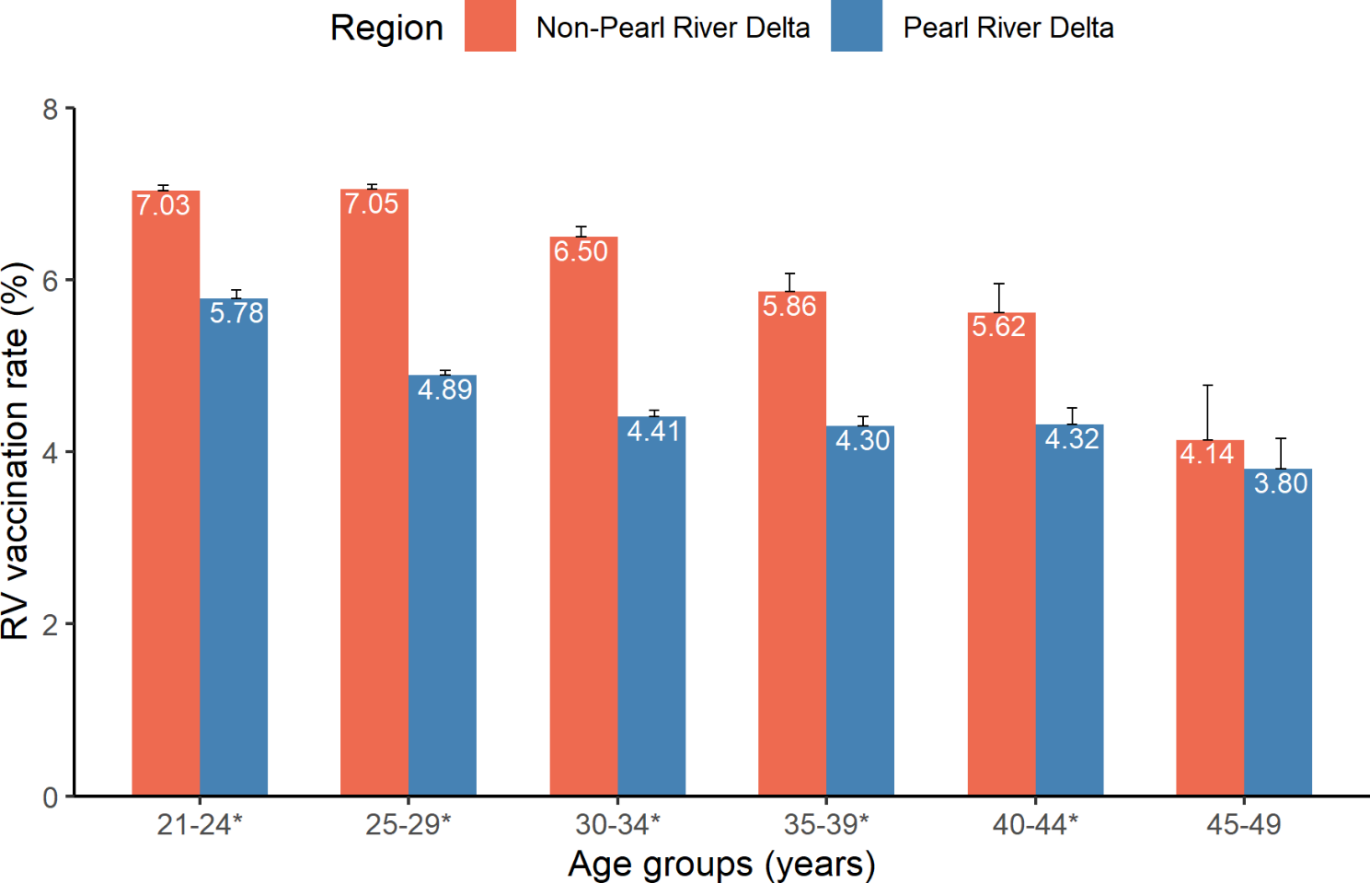
Age-specific prevalence of RV vaccination in different residential regions, 2014–2019, Guangdong. *The age group with significant differences in RV vaccination rate between those in the Non-Pearl River Delta region and those in the Pearl River Delta region

Among the 2,409,137 reproductive-aged women, 141,047 (5.85%, 95% *CI*:5.83–5.88%) women reported a history of immunization for RV. We then analyzed the prevalence of RV vaccination, stratified by age group and residential region. Women living in cities in the Pearl River Delta region had lower RV vaccination rates than those living in other cities (Fig. 3). The seropositivity of RV IgG antibodies among those having no RV vaccination history was 76.21% (95% *CI*:76.16–76.26%). In the multivariate logistic regression analysis based on data from those who did not receive the RV vaccination (Table 4), factors such as being elderly, having a higher educational level, being of other ethnicities, living in the Pearl River Delta region, and having a history of gynecological diseases and STIs were all positively associated with the prevalence of naturally acquired RV infection.

**Table 4:** Association between sociodemographic, gynecological, and obstetric characteristics and the prevalence of RV infection in natural ways

Characteristics	Total	Positive	Seropositivity (95% *CI*)	c*OR* (95% CI)	a*OR* (95% CI)
**All participants**	2,268,090	1,728,470	76.21 (76.16–76.26)	-	-
**Age groups, years**					
21–24	584,978	416,460	71.19 (71.09–71.29)	1.00	1.00
25–29	1,027,430	789,981	76.89 (76.82–76.96)	1.35 (1.34–1.36)	1.16 (1.15–1.17)
30–34	408,776	325,264	79.57 (79.46–79.68)	1.58 (1.56–1.59)	1.21 (1.20–1.23)
35–39	170,970	136,133	79.62 (79.43–79.81)	1.58 (1.56–1.60)	1.18 (1.16–1.20)
40–44	60,219	47,782	79.35 (79.02–79.67)	1.55 (1.52–1.59)	1.19 (1.16–1.22)
45–49	15,717	12,850	81.76 (81.15–82.36)	1.81 (1.74–1.89)	1.30 (1.24–1.36)
**Education**					
Primary school or below	48,318	34,128	70.63 (70.22–71.04)	1.00	1.00
Junior high school	631,106	448,012	70.99 (70.89–71.08)	1.02 (1.00-1.04)	0.97 (0.95–0.99)
Senior high school	544,309	415,002	76.24 (76.14–76.34)	1.33 (1.31–1.36)	1.10 (1.07–1.12)
College or higher	783,603	634,075	80.92 (80.84-81.00)	1.76 (1.73–1.80)	1.20 (1.18–1.23)
**Occupation**					
Farmers	462,494	331,505	71.68 (71.57–71.79)	1.00	1.00
Workers	512,541	387,052	75.52 (75.41–75.62)	1.22 (1.21–1.23)	0.96 (0.95–0.97)
Others	955,359	751,957	78.71 (78.64–78.78)	1.46 (1.45–1.47)	0.97 (0.97–0.98)
**Ethnicity**					
Han	2,165,046	1,648,736	76.15 (76.10–76.20)	1.00	1.00
Others	21,057	17,062	81.03 (80.49–81.56)	1.34 (1.29–1.38)	1.24 (1.20–1.29)
**Household registration**					
Rural	1,663,625	1,244,572	74.81 (74.75–74.87)	1.00	1.00
Urban	602,474	482,244	80.04 (79.95–80.13)	1.35 (1.34–1.36)	1.00 (0.99–1.01)
**Region**					
Non-Pearl River Delta	1,113,635	788,743	70.83 (70.75–70.90)	1.00	1.00
Pearl River Delta	1,154,455	939,727	81.40 (81.34–81.46)	1.80 (1.79–1.81)	1.76 (1.75–1.78)
**History of pregnancy**					
No	1,345,567	1,011,408	75.17 (75.10-75.23)	1.00	1.00
Yes	910,607	707,818	77.73 (77.65–77.81)	1.15 (1.15–1.16)	1.01 (1.00-1.01)
**History of gynecological diseases**					
No	2,175,827	1,654,035	76.02 (75.97–76.07)	1.00	1.00
Yes	75,943	61,297	80.71 (80.43–80.99)	1.32 (1.30–1.34)	1.07 (1.05–1.10)
**History of STIs**					
No	2,264,620	1,725,654	76.20 (76.15–76.25)	1.00	1.00
Yes	3470	2816	81.15 (79.81–82.44)	1.34 (1.24–1.46)	1.11 (1.01–1.22)

RV: Rubella virus; STIs: Sexually transmitted infections; *CI*: Confidence interval; c*OR*: Crude odds ratio; a*OR*: Adjusted odds ratio

## Discussion

Maternal preconception screening, early recognition, and treatment are key factors in the management of TOX, CMV, and RV infections during pregnancy. Based on the NFPHEP conducted in both rural and urban areas of Guangdong province between 2014 and 2019, this population-based study presents the epidemiological profile and associated influential factors of TOX, CMV, and RV infections acquired naturally. Using more than 2.4 million samples, the findings of this study are useful for reflecting the reality of the preconception of TORCH infections among women with a desire for fertility in Southern China and ascertaining target populations for intervention to reduce congenital infection.

In the present study, the overall prevalence of TOX IgG (3.20%) among women with fertility desire remained relatively low compared to that in other countries such as the United States (9.7–15.0%) [21, 27], Southeast Asia (5.3–39.7%) [28] and Ethiopia (77.7–85.1%) [29]. However, this was higher than that in most other provinces in China, as TOX-IgG seropositivity among reproductive-aged women has previously been reported to be 2.27% in Guangxi [30] and 1.61% in Liaoning [31]. In addition, geographical variation in TOX seropositivity in Guangdong province was observed in this study, and women living in the Pearl River Delta region had a significantly higher risk of TOX infection. This might be explained by the shift in population migration to more developed regions in the quest for job and study opportunities [17], and eating habits shifting to a larger proportion of undercooked seafood and vegetables. Consistent with previous studies [32, 33], we also found the lowest risk of TOX infection among women aged 25–29 years and an increasing infection risk with increasing age. Having a rural household registration and a lower educational level are risk factors for TOX infection, which might result from higher exposure to contaminated soil or water and limited awareness about the disease [34, 35]. Our study also found that women with a history of pregnancy had a higher risk of TOX than non-pregnant women, suggesting that alterations in the immune mechanisms inherent to gestation might make women more vulnerable to this pathogen [22, 36]. At the same time, since approximately 97% of women with a desire for fertility are susceptible to TOX, the need for health education and targeted prevention strategies is underscored to prevent TOX infection during pregnancy.

Unlike TOX, CMV IgG antibodies provide only partial protection [37]. Infants born to mothers with immunity to CMV may acquire congenital CMV infection because of the recurrence of latent infections or reinfection with new strains during pregnancy [38]. Our results showed that the overall seropositivity rate of CMV IgG antibodies among women with fertility desire in Guangdong was 77.67%, which is much higher than the results of a study conducted in 2010–2012 reporting a seropositivity for CMV IgG antibodies of 39.9% among rural women in the same province [15]. This inconsistency implies that comprehensive preconception screening for both urban and rural women is vital to more precisely estimate the prevalence of CMV and identify women with possible infection before pregnancy as much as possible. The higher prevalence of CMV infection among urban women in this study further emphasizes the reproductive health of urban women. The prevalence of CMV IgG increased with age, which is in accordance with previous studies [39, 40] and may be explained by the fact that accumulated exposure during a lifetime leads to an increased probability of infection. The disparity of CMV prevalence between Han people and other ethnicities might be associated with the level of health knowledge and high-risk behavior related to some ethnic customs [41, 42]. Moreover, the CMV infection rate was higher in the Pearl River Delta region than in other regions (89.89% vs. 65.27%; a*OR* = 4.45, 95% *CI*:4.41–4.49). This may be explained by the fact that close contact within a population facilitates the transmission of CMV, as the population density in the Pearl River Delta is much higher than that elsewhere in Guangdong. The presence of CMV IgG antibodies is significantly correlated with a history of pregnancy, gynecological diseases, and STIs, emphasizing the important role of sexual transmission in fueling CMV prevalence [43]. Intervention programs targeting spouses, including health education and CMV monitoring, should not be disregarded.

CRS is a frequent cause of birth defects in countries where RV is endemic, and the problem in the Asian region is prominent [44]. It is estimated that there are approximately 5.1 CRS cases per 100,000 live births in China [45]. Maternal immunity to RV before pregnancy protects newborns from CRS [46]; therefore, information on the seropositivity of RV IgG among women who desire fertility helps estimate the immune protection level and CRS risk. Earlier studies in China reported that the seropositivity rates of RV IgG among rural women were 81.0% in Guangdong [16], 78.91% in Hubei [47], and 91.28% in Hainan [48]. This study presented a self-reported RV vaccination rate of 5.85% in Guangdong during 2014–2019, and 76.21% of fertile women desired to acquire RV IgG antibodies by natural infection. Although the measles-mumps-rubella vaccine (MMR) was introduced into the Expanded Programme on Immunization (EPI) by the Chinese government in 2008 [49], the birth cohort we studied was born before the EPI, and no catch-up program for reproductive-aged women was conducted. A large proportion of women are still susceptible to RV infection or develop immunity through natural infection; therefore, continuous attention to the risks associated with RV infection is needed. Consistent with previous reports [50, 51], this study found that women with an educational level of college or higher had the highest RV IgG antibody prevalence. This might be explained by the fact that well-educated women are more likely to contract the virus in various environments and lifestyle patterns as they are often more mobile [52]. Notably, a stratified analysis of residential region revealed that women living in the Pearl River Delta region had significantly lower RV vaccination rates. This might be explained by limited access to public services in such a densely populated environment and the relatively low awareness of healthcare for vulnerable women, such as migrants. Several studies conducted in developed countries have shown that the strategy of catch-up vaccination for older children, adolescents, and reproductive-aged women is beneficial for eliminating congenital rubella [53, 54]. Therefore, continuous screening for RV susceptibility among women with fertility desires and supplementary inoculation of high-risk populations may also be meaningful in China.

This study may be the largest and most recent investigation on the prevalence of preconception TORCH infections in Southern China, with high response rates among women with a desire for fertility in both urban and rural areas. However, the present study had some limitations. First, information on the history of RV vaccination and gynecological and obstetric characteristics was self-reported, which could have led to recall bias. Second, although several sociodemographic, gynecological, and obstetric factors were considered in this study, the association between TOX prevalence and other confounders, such as dietary habits and exposure to cats, was not evaluated. Third, other TORCH pathogens such as HSV, HBV, and syphilis were not included in this study.

In conclusion, the overall prevalence of TOX-IgG among women with a desire for fertility in Southern China remains relatively low, but the absolute number of women infected with TOX and the susceptible population remains large. A large proportion of women (approximately 77%) acquire IgG antibodies against CMV and RV through natural infection. We also observed a relatively low RV vaccination rate, particularly in the Pearl River Delta region. The epidemics of TOX, CMV, and RV infections were significantly more severe in the Pearl River Delta region than in other regions. In addition, demographic (including age, educational level, occupation, and ethnicity), gynecological, and obstetric characteristics were potentially associated with the preconception of TORCH infections, but the association varied across pathogens. Due to the large population density and limited access to health resources per capita, a comprehensive and targeted prevention strategy, including preconception screening, health education, and catch-up immunization, is required to curb further TORCH transmission and reduce the risk of congenital infection.

### Electronic supplementary material

Below is the link to the electronic supplementary material.

**Additional File 1:** Figure and Table

## Data Availability

Data are available upon reasonable request via email to the corresponding author (sysuhanlu@126.com).
