# Supplementary material for: Prevalence of preconception TORCH infections and its influential factors: evidence from over 2 million women with fertility desire in southern China
Source: BMC Womens Health. 2023 Aug 10;23:425. doi: 10.1186/s12905-023-02560-4 (PMC10416474; doi:10.1186/s12905-023-02560-4)
Supplement: Supplementary file 1 — Additional File 1: Figure and Table [file 12905_2023_2560_MOESM1_ESM.docx]

**Supplementary Material**

**Prevalence of preconception TORCH infections and its influential factors: Evidence from over 2 million women with fertility desire in southern China**

Lu Han, Rui Li, Wenxue Xiong, Yang Hu, Jiabao Wu, Xiaohua Liu, Hua Nie, Weibing Qin, Li Ling, Mingzhen Li

**Fig S1.** Flowchart of Participant Selection

**Table S1.** Characteristics of 2,268,090 participants who reported no history of RV vaccination, Guangdong, 2014-2019, *n* (%)

This supplementary material has been provided by the authors to give readers additional information about their work.


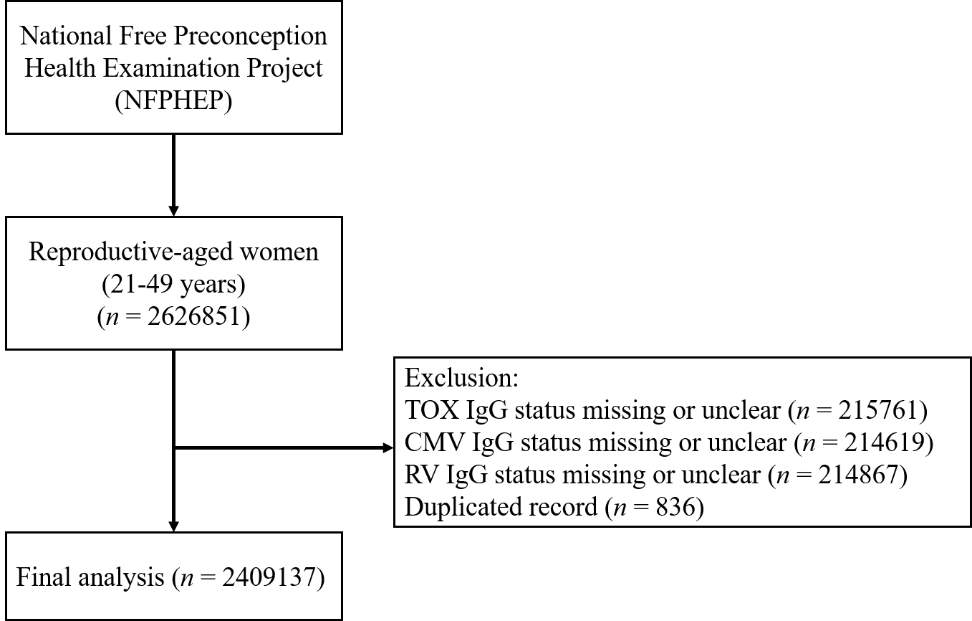


**Fig S1.** **Flowchart of Participant Selection.** TOX: Toxoplasma gondii; CMV: Cytomegalovirus; RV: Rubella virus; IgG: Immunoglobulin G.

**Table S1. Characteristics of 2,268,090 participants who reported no history of RV vaccination, Guangdong, 2014-2019, *n* (%)**

|  | 2014-2019 | 2014 | 2015 | 2016 | 2017 | 2018 | 2019 |
| --- | --- | --- | --- | --- | --- | --- | --- |
| **All participants** | 2268090 | 402428 | 360487 | 459503 | 413258 | 365377 | 267037 |
| **Age groups, years** | |  |  |  |  |  |  |
| 21-24 | 584978 (25.79) | 135424 (33.65) | 114925 (31.88) | 99529 (21.66) | 97293 (23.54) | 82027 (22.45) | 55780 (20.89) |
| 25-29 | 1027430 (45.30) | 188937 (46.95) | 178367 (49.48) | 178075 (38.75) | 180706 (43.73) | 171849 (47.03) | 129496 (48.49) |
| 30-34 | 408776 (18.02) | 57286 (14.24) | 49083 (13.62) | 92501 (20.13) | 80193 (19.41) | 72749 (19.91) | 56964 (21.33) |
| 35-39 | 170970 (7.54) | 16521 (4.11) | 13662 (3.79) | 61245 (13.33) | 36420 (8.81) | 25793 (7.06) | 17329 (6.49) |
| 40-44 | 60219 (2.66) | 3795 (0.94) | 3795 (1.05) | 24004 (5.22) | 14038 (3.40) | 9268 (2.54) | 5319 (1.99) |
| 45-49 | 15717 (0.69) | 465 (0.12) | 655 (0.18) | 4149 (0.90) | 4608 (1.12) | 3691 (1.01) | 2149 (0.80) |
| **Education** |  |  |  |  |  |  |  |
| Primary school or below | 48318 (2.13) | 12482 (3.10) | 8612 (2.39) | 8889 (1.93) | 7853 (1.90) | 6682 (1.83) | 3800 (1.42) |
| Junior high school | 631106 (27.83) | 150196 (37.32) | 114543 (31.77) | 121543 (26.45) | 109051 (26.39) | 79536 (21.77) | 56237 (21.06) |
| Senior high school | 544309 (24.00) | 98669 (24.52) | 85777 (23.79) | 99292 (21.61) | 101029 (24.45) | 92004 (25.18) | 67538 (25.29) |
| College or higher | 783603 (34.55) | 115104 (28.60) | 120348 (33.38) | 171956 (37.42) | 139472 (33.75) | 132917 (36.38) | 103806 (38.87) |
| Not available | 260754 (11.50) | 25977 (6.46) | 31207 (8.66) | 57823 (12.58) | 55853 (13.52) | 54238 (14.84) | 35656 (13.35) |
| **Occupation** |  |  |  |  |  |  |  |
| Farmers | 462494 (20.39) | 118297 (29.40) | 82400 (22.86) | 87899 (19.13) | 73611 (17.81) | 61349 (16.79) | 38938 (14.58) |
| Workers | 512541 (22.60) | 92964 (23.10) | 82525 (22.89) | 97212 (21.16) | 100313 (24.27) | 77310 (21.16) | 62217 (23.30) |
| Others | 955359 (42.12) | 157400 (39.11) | 154299 (42.80) | 206028 (44.84) | 168883 (40.87) | 152390 (41.71) | 116359 (43.57) |
| Not available | 337696 (14.89) | 33767 (8.39) | 41263 (11.45) | 68364 (14.88) | 70451 (17.05) | 74328 (20.34) | 49523 (18.55) |
| **Ethnicity** |  |  |  |  |  |  |  |
| Han | 2165046 (95.46) | 392148 (97.45) | 348186 (96.59) | 437954 (95.31) | 393098 (95.12) | 342809 (93.82) | 250851 (93.94) |
| Others | 21057 (0.93) | 3746 (0.93) | 3397 (0.94) | 3885 (0.85) | 3721 (0.90) | 3522 (0.96) | 2786 (1.04) |
| Not available | 81987 (3.61) | 6534 (1.62) | 8904 (2.47) | 17664 (3.84) | 16439 (3.98) | 19046 (5.21) | 13400 (5.02) |
| **Household registration** |  |  |  |  |  |  |  |
| Rural | 1663625 (73.35) | 312981 (77.77) | 275403 (76.40) | 315513 (68.66) | 298011 (72.11) | 263825 (72.21) | 197892 (74.11) |
| Urban | 602474 (26.56) | 89444 (22.23) | 85013 (23.58) | 142826 (31.08) | 114494 (27.71) | 101552 (27.79) | 69145 (25.89) |
| Not available | 1991 (0.09) | 3 (0.00) ^a^ | 71 (0.02) | 1164 (0.25) | 753 (0.18) | 0 (0.00) | 0 (0.00) |
| **Region** |  |  |  |  |  |  |  |
| Non-Pearl River Delta | 1113635 (49.10) | 209333 (52.02) | 181719 (50.41) | 215852 (46.98) | 209769 (50.76) | 173799 (47.57) | 123163 (46.12) |
| Pearl River Delta | 1154455 (50.90) | 193095 (47.98) | 178768 (49.59) | 243651 (53.02) | 203489 (49.24) | 191578 (52.43) | 143874 (53.88) |

NFPHEP: National Free Preconception Health Examination Project

^a^ The proportion is less than 0.01 and has been rounded.
